# Editing of the human TRIM5 gene to introduce mutations with the potential to inhibit HIV-1

**DOI:** 10.1371/journal.pone.0191709

**Published:** 2018-01-26

**Authors:** Caroline Dufour, Alix Claudel, Nicolas Joubarne, Natacha Merindol, Tara Maisonnet, Nasser Masroori, Mélodie B. Plourde, Lionel Berthoux

**Affiliations:** Laboratory of Antiviral Immunity, Department of Medical Biology, Université du Québec à Trois-Rivières, Trois-Rivières, Québec, Canada; Scripps Research Institute, UNITED STATES

## Abstract

The type I interferon (IFN-I)-inducible human restriction factor TRIM5α inhibits the infection of human cells by specific nonhuman retroviruses, such as N-MLV and EIAV, but does not generally target HIV-1. However, the introduction of two aminoacid substitutions, R332G and R355G, in the human TRIM5α (huTRIM5α) domain responsible for retroviral capsid recognition leads to efficient HIV-1 restriction upon stable over-expression. CRISPR-Cas-based approaches to precisely edit DNA could be employed to modify *TRIM5* in human cells. Toward this aim, we used a DNA transfection-based CRISPR-Cas9 genome editing protocol to successfully mutate *TRIM5* to its potentially HIV-1-restrictive version by homology-directed repair (HDR) in HEK293T cells. Nine clones bearing at least one HDR-edited *TRIM5* allele containing both mutations were isolated (5.6% overall efficiency), whereas another one contained only the R332G mutation. Of concern, several of these HDR-edited clones contained on-target undesired mutations, and none had all the alleles corrected. Our study demonstrates the feasibility of editing the TRIM5 gene in human cells and identifies the main challenges to be addressed in order to use this approach to confer protection from HIV-1.

## Introduction

Viruses are obligate parasites whose success at infecting a host cell typically requires evasion from antiviral factors. In mammals, many cellular antiviral factors that can potentially interfere with the progression of viral infections have been identified. These factors can often act without external stimulation, but their expression and activity are enhanced by type I interferons (IFN-I) [1]. Among the IFN-stimulated genes (ISGs) relevant to retroviruses, the family of viruses to which HIV-1 belongs, is *TRIM5*, which encodes the cytoplasmic protein TRIM5α [2]. In humans, *TRIM5* is transcribed into 5 isoforms, among which only TRIM5α possesses antiviral activity [3]. At its C-terminus, a domain called SPRY (PRYSPRY, B30.2) determines the retrovirus targeting specificity. This domain comprises hyper-variable loops that directly interact with the N-terminal domain of capsid proteins early after entry of the retrovirus into the host cell membrane [4]. When such interactions occurs, the retrovirus is inhibited (“restricted”) through mechanisms that include destabilization of the capsid core [5], proteasomal degradation of some core components [6] and sequestration of the viral particle in TRIM5α cytoplasmic bodies [7]. huTRIM5α generally has little-to-no activity against HIV-1, but efficiently inhibits the infectivity of the nonhuman gammaretrovirus “N-tropic” murine leukemia virus (N-MLV) as well as the nonhuman lentivirus equine infectious anemia virus (EIAV) [8]. Those two viruses are typically inhibited ~10-fold (EIAV) and ~100-fold (N-MLV) by endogenous huTRIM5α, with some variation depending on the cellular context.

Several groups, including ours, have attempted to harness the antiviral power of TRIM5α in order to interfere with HIV-1. This virus is efficiently restricted (~100-fold) by some orthologs of TRIM5α found in Old World monkeys such as the Rhesus macaque TRIM5α (rhTRIM5α) [2]. However, significant sequence variation between the human and macaque orthologs preclude the possibility of using the latter one in gene therapy approaches, as this would increase the risk to elicit an immune response against the transgene in patients. Thus, all the studies have consisted in over-expressing versions of huTRIM5α designed to target HIV-1 through modifications in the SPRY domain. Some of the TRIM5α variants used were chimeric products containing small regions of rhTRIM5α in the SPRY [9, 10]. Other teams mapped with further precision the HIV-1 restriction determinants in rhTRIM5α that were absent in huTRIM5α, leading to the discovery that mutating the Arg332 residue in huTRIM5α was sufficient to inhibit HIV-1. Although initial observations [11, 12] raised the hope that single mutations at this position might inhibit HIV-1 as efficiently as rhTRIM5α did, later work made it clear that this was not the case [13].

Our laboratory explored a different approach: generating libraries of TRIM5α SPRY mutants then applying a functional screen to isolate mutants that conferred HIV-1 restriction [14, 15]. These studies identified mutations at Arg335 inhibiting HIV-1 by 5- to 10-fold. When we combined a mutation at Arg335 (R335G) with one at Arg332 (R332G), we obtained restriction levels that were higher than with either of the single mutants [14, 15]. Although not quite as restrictive as rhTRIM5α, R332G-R335G huTRIM5α efficiently inhibited the propagation of a highly pathogenic strain of HIV-1, and cells expressing the transgene had a survival advantage over unmodified cells [13].

Although R332G-R335G huTRIM5α is considered a prime candidate in HIV-1 gene therapy approaches to inhibit HIV-1 [13], using viral vectors to overexpress it in human cells is not without caveats. Indeed, the physiological effects of TRIM5α overexpression *in vivo* are not clear, considering that it is involved in innate immune responses [16, 17] and possibly in autophagy [18]. In addition, the genotoxicity of lentiviral vectors integrating in the human genome is still poorly predictable. In the longer term, it would thus be desirable to be able to introduce mutations in the endogenous human *TRIM5* by genome editing. The most advanced approach toward this aim consists in inducing double strand DNA breaks through the use of the Clustered Regularly Interspaced Short Palindromic Repeats (CRISPR) with CRISPR-associated protein 9 (CRISPR-Cas9) system, and simultaneously providing a single-stranded “donor DNA” to serve as a template for homology-directed repair (HDR) [19, 20]. In this report, we use a simple, DNA transfection-based protocol to introduce the various components required into a highly transfection-permissive human cell line, HEK293T. We successfully mutate Arg332 and Arg335 of *TRIM5* in these cells, although undesired mutations are also found and no cell clone could be isolated that had all *TRIM5* alleles bearing the corrections.

## Results

### HEK293T cells provide a poor cellular environment for TRIM5α antiviral activity

**Fig 1:**
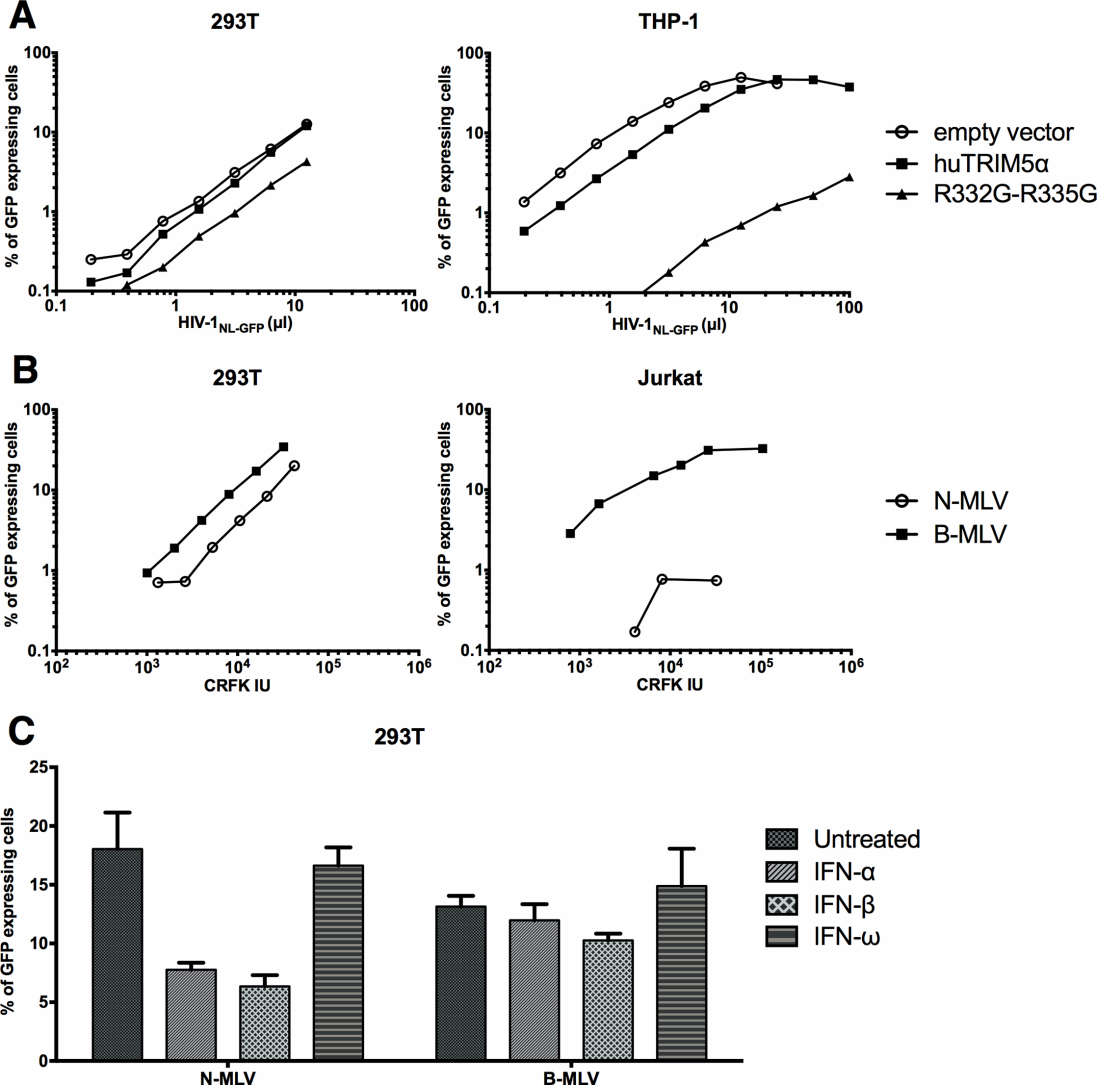
Restriction by exogenous or endogenous TRIM5α is inefficient in HEK293T cells. (A) HEK293T and THP-1 cells were retrovirally transduced with WT huTRIM5α, R332G-R335G huTRIM5α or with the “empty” vector as indicated. Untransduced cells were eliminated by hygromycin treatment, and the cell populations were then challenged with increasing amounts of the HIV-1_NL-GFP_ vector. The percentage of cells expressing GFP was then determined by FACS.(B) HEK293T Jurkat cells were infected with increasing amounts of N-MLV_GFP_ and B-MLV_GFP_. The amounts of virus used are expressed as multiplicities of infection (MOI) as calculated in the non-restrictive CRFK cells. The percentage of infected cells was determined by FACS. For N-MLV in Jurkat cells, only the 3 virus doses that yielded detectable infections are shown. (C) HEK293T cells were treated with IFN-α, IFN-β or IFN-ω for 16 h prior to a single-dose infection with N-MLV_GFP_ and B-MLV_GFP_, designed to yield 10–20% infected cells in the absence of IFN-I. The percentage of infected cells was determined by FACS.

HEK293T cells were chosen for this study due to their very high permissiveness to DNA transfection [21], thus allowing us to test our general strategy using simple, cost-effective methods of proven robustness. Perhaps surprisingly, considering that HEK293T cells are commonly used to produce retroviral vectors, the capacity of TRIM5α to inhibit retroviruses in this cell line is poorly known. We found one study in which the authors stably transduced rhTRIM5α in HEK293 cells, leading to a ~5-fold decrease in permissiveness to a GFP-expressing HIV-1 vector [22]. This level of restriction is more than 10-fold lower than what is usually found in other human cell lines in similar experimental settings [14, 17]. To evaluate the potential of HEK293T cells as a cellular model for R332G-R335G huTRIM5α-mediated HIV-1 restriction, we retrovirally transduced the gene in this cell line and (as a control) in the monocytic cell line THP-1. The unmutated (wild-type, WT) version of huTRIM5α, which restricts HIV-1 at very low levels, was used as an additional control. Untransduced cells were killed by antibiotic treatment. Then, the cells were challenged with an HIV-1 vector expressing GFP, and the percentage of infected cells was determined by FACS. We found that in THP-1 cells, over-expression of R332G-R335G huTRIM5α but not WT huTRIM5α reduced permissiveness to HIV-1 by ~100-fold; however, the magnitude of restriction was much smaller (~3-fold) in HEK293T (Fig 1A). These results suggest that HEK293T cells provide a suboptimal environment for HIV-1 restriction by over-expressed TRIM5α. To investigate whether this observation extended to the endogenous TRIM5α in HEK293T, we infected the parental cells with increasing amounts of N-MLV_GFP_ or with the huTRIM5α-insensitive B-MLV_GFP_. As a control, we used the same vectors to infect Jurkat cells. When the amounts of vectors used were normalized for their infectious power in a non-restrictive cell line (CRFK cells), we found that as expected, N-MLV_GFP_ was highly restricted in Jurkat cells, compared with B-MLV_GFP_ (Fig 1B). In contrast, N-MLV_GFP_ was only 2- to 3-times less infectious than B-MLV_GFP_ in HEK293T cells (Fig 1B). Such poor restriction levels could be explained by low TRIM5α expression levels. In order to boost *TRIM5* transcription, we treated the cells with IFN-α, IFN-β or IFN-ω and then infected them with the two MLV-based vectors (Fig 1C). We observed that treatment with IFN-α and IFN-β, but not IFN-ω, slightly decreased (2-fold) the infectivity of the N-MLV vector and had no significant effect on B-MLV. Thus, IFN-I treatments moderately enhanced the restriction of N-MLV but failed to induce restriction levels consistent with what is generally found for this murine virus in human cells. We conclude that the potential for TRIM5α-mediated restriction is abnormally low in HEK293T cells, irrespective of whether TRIM5α is expressed endogenously or exogenously and irrespective of the targeted virus.

### Strategy for the mutagenesis of *TRIM5* by HDR

**Fig 2:**
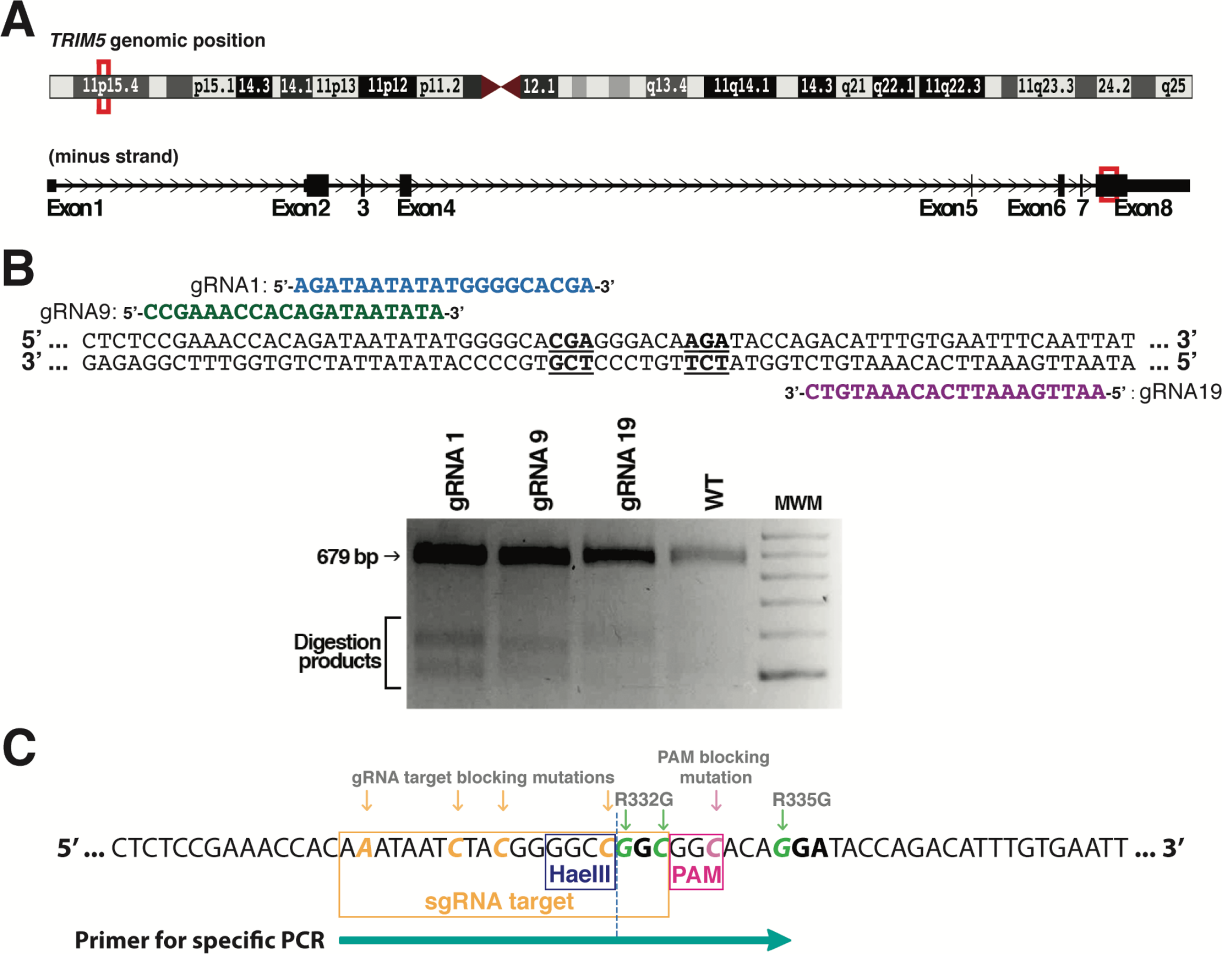
Design of the gRNA and donor ssODN for the HDR-mediated editing of *TRIM5*. (A) *TRIM5* localization on chromosome 11 (top), and Arg332-Arg335 localization in exon 8 of the gene (bottom). (B) Top panel: position of the three gRNAs (gRNA1, 9 and 19) designed to target the Arg332-Arg335 region. The two arginine codons are underlined and in bold. Bottom panel: Surveyor assay following the transfection of HEK293T cells with CRISPR-Cas9 plasmids expressing one of the three gRNAs. WT DNA from untransfected cells was used as a control. (C) HDR donor DNA mutagenesis strategy. 8 substitutions were present, including three nonsilent substitutions to mutate Arg332 and Arg335 into Gly (green), one silent mutation to disrupt the PAM sequence (pink), and four silent mutations in the sequence targeted by gRNA1 (orange). The HaeIII restriction site created as a result of one of the silent substitutions is indicated, as is the position of the primer used in specific PCR screening.

Human *TRIM5* is found on chromosome 11p15.4 and the region of the gene encoding the SPRY domain is present in exon 8 (Fig 2A). We searched for DNA loci close to the codons for Arg332 and Arg335 that would be potential targets for CRISPR-Cas9-mediated double-strand cleavage. CRISPR guide RNAs (gRNAs) were designed for the 3 potential target sites that were nearest to the two codons to be mutated (Fig 2B). CRISPR plasmids expressing Cas9 along with one of the designed gRNAs were transfected in human embryonic kidney 293T cells (HEK293T), and a Surveyor assay was performed to test the capacity of the three gRNAs to target the endogenous TRIM5 gene. Results showed that all three gRNAs were competent (Fig 2B). Because gRNA1 induces a cleavage that is closest to the targeted codons (right before the first nt of Arg332), the rest of the project was carried out with this gRNA. The HDR donor DNA consisted of a single-stranded oligodeoxynucleotide (ssODN) that was 200 nt long and antisense relative to the gRNA, in keeping with published methods [19, 23]. The central section of this ssODN containing the mutations introduced is shown in Fig 2C (depicted in the same orientation as the TRIM5 mRNA for clarity purposes). In addition to the mutations substituting arginine residues into glycine at positions 332 and 335, we included 4 silent mutations in the region recognized by the gRNA and 1 more silent mutation in the protospacer adjacent motif (PAM), amounting to a total of 7 substitutions expected to prevent the cleavage of HDR-corrected DNA by Cas9. One of the mutations also created a HaeIII cut site for convenient downstream screening of the cell clones obtained.

### Isolation of *TRIM5*-edited HEK293T clones

**Fig 3:**
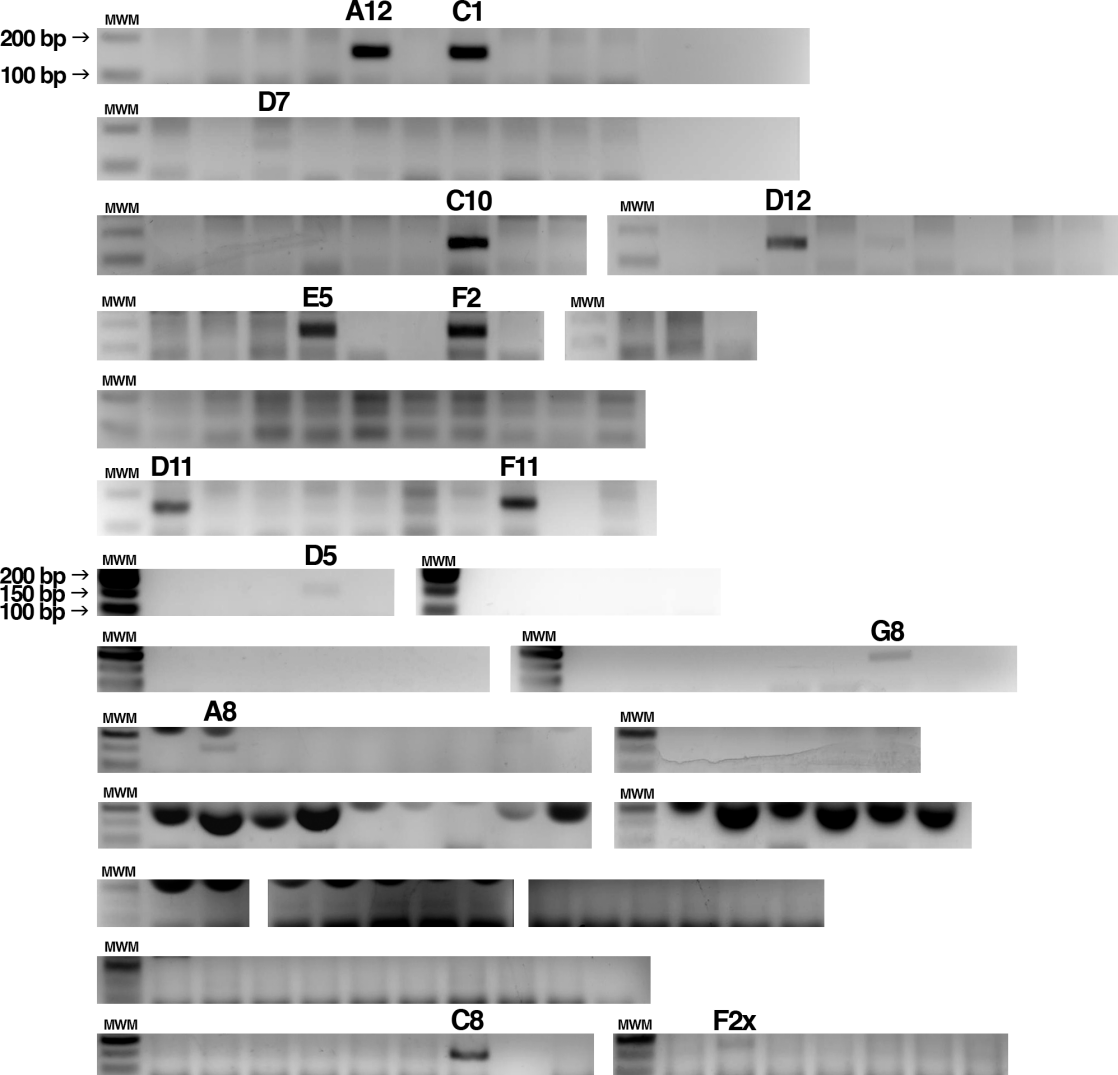
Pre-screening of isolated clones by specific PCR. Following 20 days of growth, 161 isolated HEK293T clones were screened for HDR-edited TRIM5 gene by PCR using a primer specific for the mutated *TRIM5*. 14 clones that passed this pre-screen step are indicated by their names. MWM, molecular weight marker.

We transiently transfected a plasmid (pX459) expressing Cas9 and the gRNA1 into HEK293T cells, along with the ssODN. Single-cell clones were then isolated by limiting dilution. We screened 161 clones at random for the presence of HDR-modified alleles by specifically amplifying the mutated TRIM5 sequence using a primer whose sequence is indicated in Fig 2C. As shown in Fig 3, 14 clones showed a positive signal (of varying intensity) in this assay. One of the clones, F2X, yielded a band whose size seemed bigger compared to another positive clone (C8) analyzed on the same gel.

**Fig 4:**
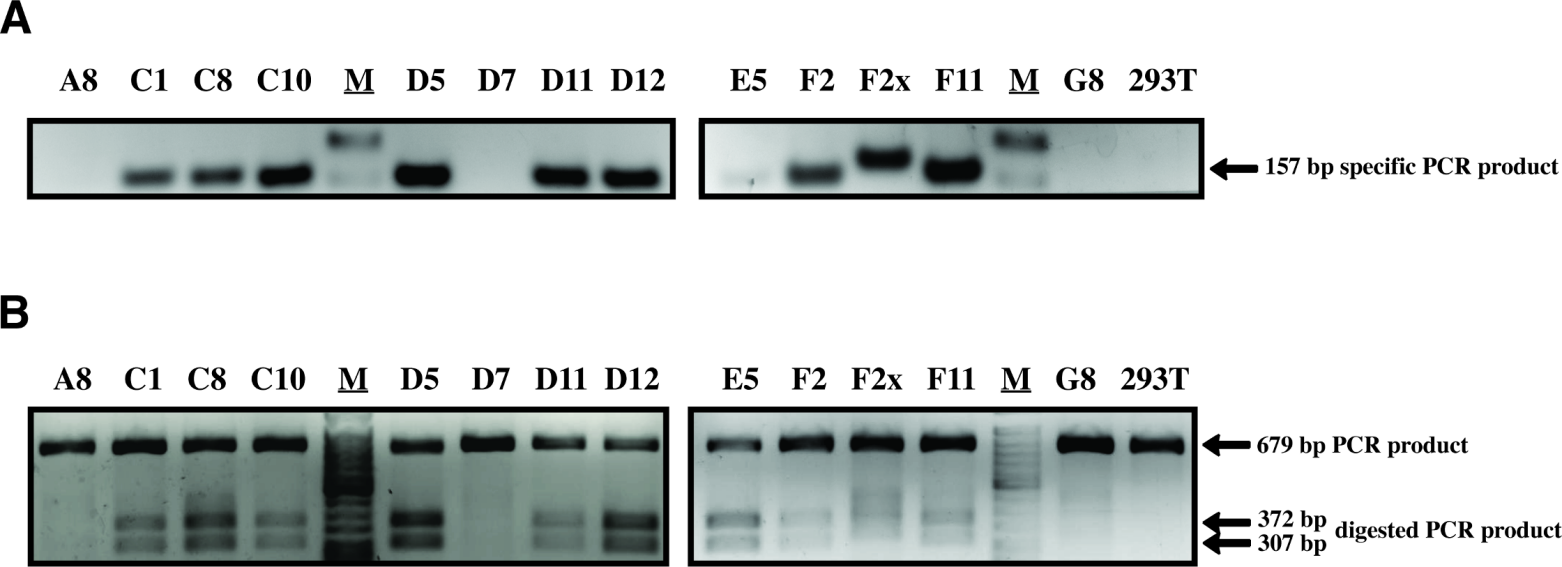
Identification of HDR-edited clones. (A) Mutation-specific PCR was performed on 13 clones showing a positive signal in the pre-screen. Untransfected HEK293T cells were used as a control. M, molecular weight marker. (B) Non-specific PCR of the targeted region followed by HaeIII digestion. The expected sizes of the digested PCR products are shown on the right. The full-length gels are available on the FigShare public repository (see “Availability of data” section).

All these clones (minus A12, which did not survive) were re-analyzed using the same specific PCR assay and also using a second assay in which the targeted region is amplified and then digested by HaeIII, which cuts at a site created by one of the silent mutations (see Fig 1C). In the latter assay, amplification was done using primers that bind outside the 200 nts corresponding to the donor ssODN in order to insure that the ssODN was not inadvertently detected. Fig 4 shows a positive signal for 10 of these clones in both assays. The three remaining clones were negative in both assays.

### Genotype analysis

**Fig 5:**
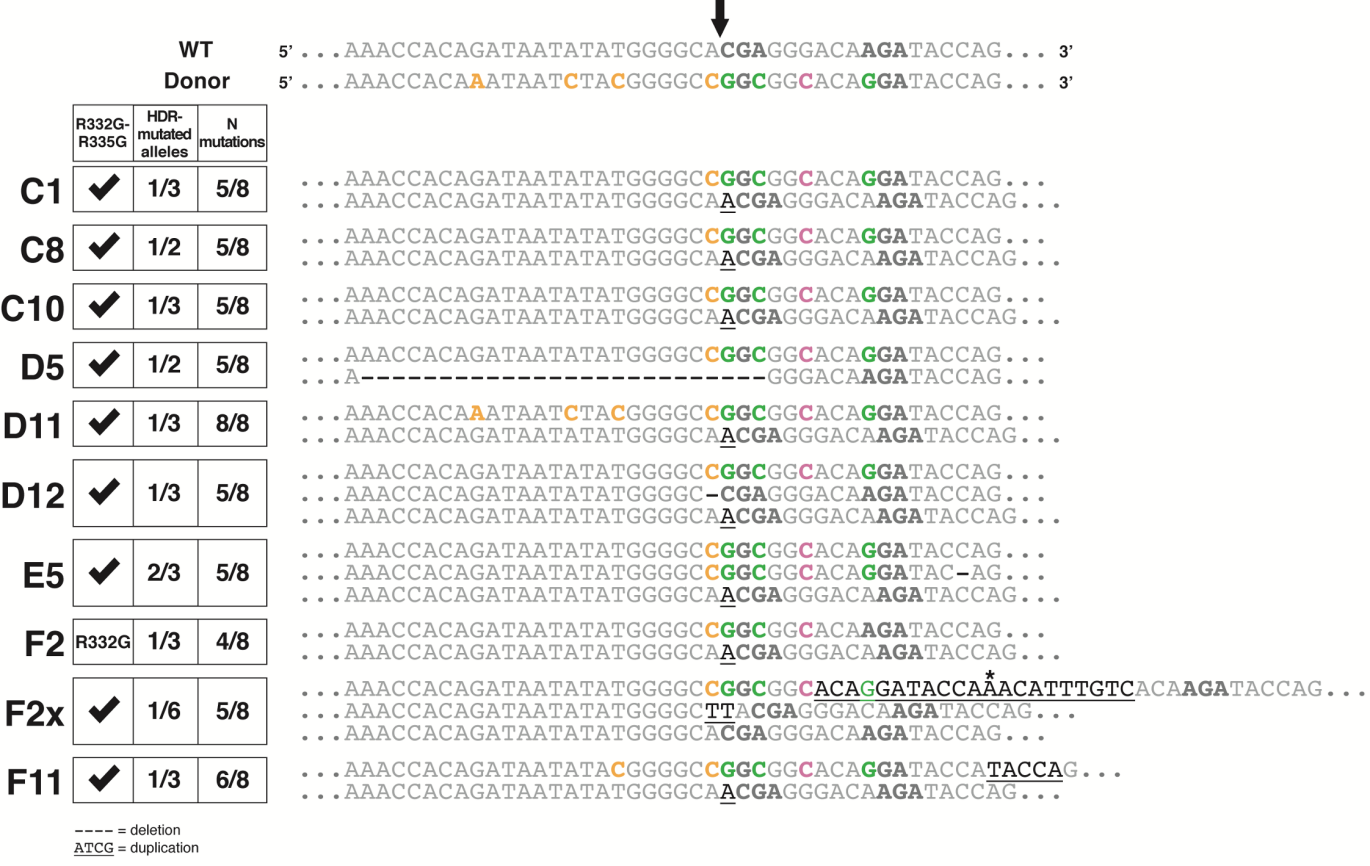
Deep sequencing analysis of *TRIM5* editing in 10 screened clones. The ~200-nt HDR-targeted *TRIM5* region was amplified by PCR and the PCR products were then analyzed by Illumina MiSeq sequencing. The alignment shown includes the targeted locus for each allele of the 10 clones, in comparison with the WT sequence and with the expected HDR-mutated sequence (top 2 lines). The codons for aa 332 and 335 are in bold. The Cas9 cleavage site on the WT sequence is shown at the top (arrow). Expected substitutions are shown in green (R332G/R335G), in orange (silent mutations in the gRNA target sequence) and in pink (silent mutation in the PAM). Duplications/insertions are underlined whereas deletions are represented by dashes. The star indicates the position of an unexpected substitution within a duplicated region in one allele of F2X. On the left is a table summarizing the results obtained for each clone: presence of the two therapeutic mutations R332G/R335G, proportion of *TRIM5* alleles modified by HDR and the proportion of the expected substitution mutations in the HDR-edited alleles. Note that only one clone (D11) has an allele containing all the desired mutations and that most of the non-HDR-edited alleles contain indels at the cleavage site.

The 10 clones showing indications of HDR-mediated editing were then subjected to PCR using the same primers that were also utilized in the HaeIII screen described above. PCR products were analyzed by deep sequencing, and a color-coded alignment of the results is shown in Fig 5. The HEK293 cells and their derivatives are pseudotriploid [24] and accordingly, we found that two clones had two *TRIM5* alleles and seven were triploid. F2X seemed to possess 6 *TRIM5* alleles, a finding that is discussed below. For each clone, both desired mutations at Arg332 and Arg335 were present on one allele or more, with the exception of F2 which only had the Arg332 mutation. However, only one clone (D11) contained an allele with all 8 substitutions present. The HDR-generated alleles in the other cell clones generally contained the expected mutations in the PAM-proximal side of the cleavage site, with the exception of F2 which lacked the A-to-G mutation required to introduce the R335G change. All HDR-generated alleles had the A-to-C silent mutation creating the HaeIII restriction site at the first nucleotide upstream of the cleavage site on the PAM-distal side. This is consistent with the fact that all clones that were found to be positive in the specific PCR screen were also positive in the HaeIII assay (Fig 4). Strikingly, for the three other substitution mutations in the PAM-distal region, only one clone (D11) had all of them whereas another one (F11) had only one. It would be tempting to conclude that HDR is biased so that mutations were more likely to be incorporated in the PAM-proximal side of the cut, and other teams have reported such imbalances in the conversion rate [25]. On the other hand, our specific PCR screen requires a successful amplification using a primer whose 3’ half is complementary to the PAM-proximal region (Fig 2C), thus creating a bias toward the detection of mutated DNA containing the expected mutations in this PAM-proximal region.

Some HDR-generated alleles showed additional, unexpected mutations. In E5, one of the two HDR alleles had a one-nt deletion 16 nts from the cleavage site in the PAM-proximal region. In F11, the HDR allele had a 5 nt (TACCA) duplication in the same region. F2X showed an intriguing genotype: firstly, we found that the HDR allele was present in 17% (1:6) of the amplicons, whereas 33% (2:6) were wild-type (WT) and 50% (3:6) contained a TT insertion. HEK293 cells are known to be prone to chromosomal translocations leading to a high level of variation in copy numbers [26], which might explain our findings. The HDR-generated allele in F2X had a surprising structure, with a 21 nt duplication consistent with the slower-migrating bands in Fig 3 and in Fig 4. The repeated sequence that was closest to the cleavage site had the expected mutations in the PAM and at Arg335 and also contained an additional substitution (G-to-A) that is not present in the donor ssODN, whereas the second repeat of this sequence only had the G-to-C mutation in the PAM. These HDR-generated alleles that also contained unexpected insertions/deletions (indels) are unlikely to encode functional TRIM5α, due to the frameshifts leading to premature termination (E5, F11) or due to the insertion of 7 aminoacids at a region crucial for capsid binding (F2X). The rest of the HDR-generated alleles (in C1, C8, C10, D5, D12 and one of the two HDR alleles in E5), however, may potentially encode proteins that efficiently target HIV-1.

Examination of the *TRIM5* alleles not modified by HDR in these cell clones revealed clear signs of NHEJ-induced mutations, *i*.*e*. indels at the cleavage site. 8 alleles had an A inserted at the cleavage site, whereas one of the F2X alleles had a TT inserted at the same locus. The A insertion was so prevalent that in half of the clones (C1, C10, D11, F2, F11), 2 of the 3 *TRIM5* copies were mutated by NHEJ leading to this particular mutation. Although the nature of NHEJ-directed mutations is known to vary widely depending on the gRNA used [27], such +1 insertions have been described to be prevalent as a result of Cas9 editing [28, 29]. One D5 allele had a larger, 27 nt-long deletion whereas one D12 allele had a single deletion at the cleavage site. Therefore, and with the exception of F2X, our data strongly suggest that in all cell clones in which one or two of the *TRIM5* alleles were mutated by HDR, the remaining alleles were mutated by NHEJ. This is consistent with findings published by others [19]. Furthermore, these NHEJ-generated *TRIM5* alleles are all expected to encode non-functional TRIM5α due to missense mutations in the SPRY domain and premature termination.

## Discussion

To our knowledge, this is the first attempt at using genome editing to mutate a restriction factor with the aim of conferring an innate antiviral function in human cells. Our preliminary observations indicated that restriction by TRIM5α was suboptimal in HEK293T cells. We nonetheless went ahead and attempted genome editing in this cell line, reasoning that this would provide valuable information on the efficiency and accuracy of the editing procedure itself. In order for HDR-mediated editing of a restriction factor like TRIM5α to be successful, it is presumably desirable to be able to modify both alleles of the gene in diploid cells. In addition, no additional mutations (indels especially) should be present. Our results show that on both of those points, challenges lie ahead. In all but one of the clones in which the two therapeutic mutations were present, they were found on only one allele. In almost all cases, the non-HDR-corrected allele(s) contained indels, a clear sign of NHEJ-mediated repair. It is predicted that the SPRY-truncated TRIM5α proteins resulting from the presence of such indels will interact with the full-length TRIM5α and will interfere with its targeting of incoming retroviruses, similar to the activity of natural, shorter TRIM5 isoforms [3, 30]. Another pitfall is the occurrence of unwanted on-target indels, which we observed in 3 out of 9 alleles bearing the two therapeutic mutations in this study. We did not observe HIV-1 restriction activity in any of the 9 cell clones (not shown), which likely resulted from a combination of causes including the suboptimal cellular environment and allelic heterogeneity. In conclusion, in order to obtain scarless modification of *TRIM5* by HDR, we will need to address the difficulty of achieving bi-allelic HDR-mediated editing while avoiding the advent of additional mutations in the modified alleles. Recent technological advances, including the development of a marker-free system to enrich cells in which HDR occurred [31], are likely to enhance editing efficiency as well as bi-allelic editing. The use of Cpf1 instead of Cas9 as the enzyme to generate double-strand DNA breaks might also increase the frequency of bi-allelic modifications, as this enzyme cuts at a site 17 nt distal to the PAM, and thus can potentially re-cut DNA following the incorporation of NHEJ-induced indels, unlike Cas9 [32].

## Methods

### Cells and *TRIM5* genotyping

Cell lines were obtained from J. Luban (University of Massachusetts Medical School). HEK293T and CRFK cells were maintained in DMEM medium (HyClone). THP-1 and Jurkat cells were maintained in RPMI medium (Hyclone). All culture media were supplemented with 10% fetal bovine serum (FBS) and penicillin/streptomycin (HyClone). To analyze the sequence of the targeted genomic region, cellular DNA was prepared using the Bioline genomic DNA kit (London, UK), and the *TRIM5* region encompassing the targeted locus was PCR-amplified using primers T5a_Surveyor_fwd (5’GTCCGACGCTACTGGGGTAAG) and T5a_Surveyor_rev (5’ATAATCACAGAGAGGGGCACA). The PCR product was Sanger-sequenced using the same primers. We found no variation in this region compared to the consensus sequence (NCBI Gene ID: 85363).

### Design of gRNAs and surveyor assay

The lentiviral expression vector pLentiCRISPRv2 (pLCv2) was a gift from Feng Zhang (Addgene plasmid # 52961) [33]. Three gRNAs targeting *TRIM5* were designed using the Zhang lab online software available at crispr.mit.edu. The sequences targeted are 5'AGATAATATATGGGGCACGA (gRNA1), 5'CCGAAACCACAGATAATATA (gRNA9) and 5'AATTGAAATTCACAAATGTC (gRNA19). The ODNs needed for the generation of pLCv2-based constructs targeting those sequences were designed exactly as described in published protocols [27, 33]. Sense/antisense pairs of primers were annealed and cloned into pLCv2 cut with BsmBI. To evaluate the capacity of the constructed plasmids to induce on-target indels in *TRIM5*, a surveyor nuclease assay was performed. HEK293T cells were transfected with either pLCv2-gRNA1, -gRNA9 or -gRNA19 using polyethyleneimine [34]. 3 d later, the genomic DNA was extracted from the transfected cells using the Bioline genomic DNA kit. The targeted *TRIM5* region was PCR-amplified using primers T5a_Surveyor_fwd and T5a_Surveyor_rev. PCR amplicons were heat-denatured at 95°C, and re-annealed by slow cooling to promote formation of dsDNA heteroduplexes. The heteroduplexes were then cleaved by Surveyor nuclease S provided as part of the Transgenomic Surveyor mutation detection kit (Integrated DNA Technologies, Coralville, IA), according to the manufacturer’s instructions. Digestion products were visualized by agarose gel electrophoresis.

### Design of the HDR donor DNA and *TRIM5* editing

The following *TRIM5* minus strand-derived HDR DNA was synthesized by Integrated DNA Technologies: 5’CGTCTACCTCCCAGTAATGTTTCCCTGATGTGATACTTTGAGAGCCCAGGATGCCAGTACAATAATTGAAATTCACAAATGTCTGGTATCCTGTGCCGCCGGCCCCGTAGATTATTTGTGGTTTCGGAGAGCTCACTTGTCTCTTATCTTCAGAAATGACAGCACATGAAATGTTGTTTGGAGCCACTGTCACATCAACT. Residues mutated compared to the WT *TRIM5* sequence are underlined. pX459-gRNA1 was constructed in a manner similar to pLCv2-gRNA1, by ligating the corresponding annealed gRNA1 ODN duplex into pX459 (pSpCas9(BB)-2A-Puro; Addgene #62988) [35] digested with BbsI. HEK293T cells were plated in 6-well plates at 2.7 x 10^5^ per well and transfected the next day using polyethyleneimine, with 2.5 μg of pX459-gRNA1 together with 5 μl of the HDR DNA prepared at 20 μM. When cells reached confluence, they were trypsinized and plated at 0.5 cell per well in 96-well plates, using conditioned medium. To screen the colonies for HDR-mediated *TRIM5* editing, part of the cells were lyzed in the DirectPCR Lysis reagent (Viagen Biotech, Los Angeles, USA) diluted 1:1 in proteinase K-containing water as recommended by the manufacturer. Lysis was allowed to proceed overnight at 55°C followed by heating at 85°C for 90 min to deactivate proteinase K. For the specific PCR-based screening, 5 μl of the lysed cells were subjected to PCR using primers T5a_mut_fwd (5’-AAATAATCTACGGGGCCGGCGGCACAG) and T5a_qPCR_rev (5’- CCAGCACATACCCCCAGGAT). PCR was performed for 30 cycles using the following conditions: 30 sec at 94°C, 30 sec at 61.5°C, 30 sec at 68°C. The 157-bp expected PCR product was resolved on agarose gels. For the HaeIII-based screening, lysed cells were subjected to PCR using primers T5a_Surveyor_fwd and T5a_Surveyor_rev, similar to the Surveyor assay. 10 μl of the PCR product were digested by HaeIII for 60 min at 37°C. The reaction products were analyzed using agarose gels in order to reveal the 307-bp and 372-bp bands corresponding to digested products. For MiSeq sequencing, cellular DNA was submitted to PCR using the following ODNs, which bind to DNA sequences located 10 nt outside the 200 nt-long region corresponding to the HDR: huTR5aGG_seq_FOR, 5’ACACTGACGACATGGTTCTACAATCCCTTAGCTGACCTGTTA, and huTR5aGG_seq_REV, 5’TACGGTAGCAGAGACTTGGTCTCCCCCAGGATCCAAGCAGTT. The underlined sequences are barcodes. MiSeq sequencing results were analyzed using the online tool Integrative Genomics Viewer (http://software.broadinstitute.org/software/igv/).

### Retroviral vectors production and viral challenges

To generate the HEK293T cells stably expressing WT and R332G-R335G huTRIM5α, cells were transduced with the corresponding pMIH-huTRIM5α vectors followed by hygromycin selection as described previously [36]. To produce GFP-expressing retroviral vectors, HEK293T cells were seeded in 10 cm culture dishes and transiently co-transfected with the following plasmids: pMD-G, pCNCG and pCIG3-B or pCIG3-N to produce B-MLV_GFP_ and N-MLV_GFP_, respectively; pMD-G and pHIV-1_NL-GFP_ to produce HIV-1_NL-GFP_ (see [37, 38] and references therein). Retroviral vector preparations were titrated by infecting cat CRFK cells with multiple vector doses and then calculating the MOI based on nonsaturating virus doses. For retroviral challenges, cells were seeded into 96-well plates at 10,000 cells per well and infected the following day with the GFP-expressing retroviral vectors. Cells were trypsinized at 2 d post-infection and fixed in 2.5% formaldehyde (Fisher Scientific, MA, USA). The percentage of GFP-positive cells was then determined by analyzing 1x10^4^ cells on a FC500 MPL cytometer (Beckman Coulter, CA, USA) using the CXP Software (Beckman Coulter). For infections done in presence of IFN-I, recombinant human IFN-α, IFN-β or IFN-ω (PeproTech, Rocky Hill, NJ) was added to cell cultures 16 h prior to infection and at a final concentration of 10 ng/ml.
